# Comparative study of pressure (ankle-brachial pressure index) and flow (strain gauge plethysmography and reactive hyperaemia) measurements in diagnosis of peripheral arterial disease in patients with severe aortic stenosis

**DOI:** 10.1371/journal.pone.0220510

**Published:** 2019-07-30

**Authors:** Nadjib Schahab, Rolf Fimmers, Thorsten Mahn, Christian Schaefer, Vedat Tiyerili, Georg Nickenig, Jan-Malte Sinning, Anja Stundl

**Affiliations:** 1 Department of Internal Medicine II—Cardiology, Pulmonology and Angiology, University Hospital Bonn, Bonn, Germany; 2 Institute of Medical Biometry, Informatics and Epidemiology, University Hospital Bonn, Bonn, Germany; Kurume University School of Medicine, JAPAN

## Abstract

**Background:**

The measurement of the ankle-brachial pressure index is a straightforward method for the detection of peripheral disease in the lower limbs. Only a few old studies with small numbers of patients have been conducted comparing the gold standard, ankle-brachial pressure index measurement, with strain gauge plethysmography and reactive hyperaemia for detecting peripheral arterial disease. The purpose of this study was to evaluate the feasibility and accuracy of strain gauge plethysmography values compared with the Doppler ultrasound method, ankle-brachial pressure index, in the assessment of peripheral arterial disease, especially in patients with severe aortic stenosis.

**Methods:**

221 ankle-brachial pressure index measurements and strain gauge plethysmography measurements of patients with suspected peripheral arterial disease, diagnosed peripheral arterial disease with or without aortic stenosis were compared.

**Results:**

Irrespective of aortic stenosis in patients with and without peripheral arterial disease, the resting arterial blood flow was within the normal range. In patients with aortic stenosis, the time-to-peak flow couldn’t detect peripheral arterial disease and was found to be a false negative. In patients without aortic stenosis, time-to-peak flow correlated well with the ankle-brachial pressure index for detecting peripheral arterial disease. Peak flow at 5 seconds was the one of the flow values that correlated with ankle-brachial pressure index and detected peripheral arterial disease in patients with and without aortic stenosis.

**Conclusion:**

Peak flow at 5 seconds is one of flow value that correlated well with ankle-brachial pressure index in detecting peripheral arterial disease in patients with and without aortic stenosis. Detection of peripheral arterial disease in patients with severe aortic stenosis seems to be less sensitive with flow measurements than with ankle-brachial pressure index.

## Introduction

Peripheral arterial disease (PAD) is a manifestation of generalized arteriosclerotic disease and leads to a range of clinical conditions from asymptomatic disease to critical ischemia.

The standard, quantitative, noninvasive test to assess the severity of PAD is the measurement of ankle-brachial pressure index (ABPI). It is an accurate, simple, and noninvasive measurement and considered to be the most accurate noninvasive diagnostic methode for PAD and its assessment. An ABPI below 0.9 has been widely accepted as evidence of PAD in most epidemiological studies [1].

The American College of Cardiology/American Heart Association (ACC/AHA), Society for Vascular Surgery (SVS), and European Society of Cardiology (ESC) guidelines recommend ABPI determination as the first-line noninvasive test to establish a diagnosis of PAD because of its high sensitivity and specificity [2, 3, 4].

Beside ABPI, clinical signs, 6 min Walking test, treadmeal excercise, and other nonivasive techniques have assumed an important role in detection of PAD. Both Ultrasound and plethysmography, segmental oscillography allow objective evaluation of vascular disorder by measurement of segment limb blood pressure, analysis of blood velocity disturbance, recording of digit or limb pulse waveforms. Also imaging of vascular disease, with or without Doppler flow analysis or color-flow mapping are useful methods. In patients with aortic stenosis and pre-TAVI, pre-procedural CT-angiography is clinically useful, allowing precise pre-procedural TAVI planning with accurate assessment of iliac and femoral arteries.

Only a few studies with small numbers of patients or healthy subjects have been conducted that compare ABPI measurement (as the gold standard) with Strain-gauge plethysmography—reactive hyperemia (SGP-RH) for detecting PAD [5, 6].

There are no data regarding detecting PAD with SGP-RH in patients with AS. The presence of PAD is significantly associated with increased rates of major vascular complications as well an immediate and late mortality in patients with AS undergoing TAVI. Assessment of PAD before TAVI is essential to choose an access strategy and to predict clinical results and avoide major complications.

The purpose of the present study was to examine and compare the accuracy and predictive values of SGP-RH measurements with the Doppler ultrasound method ABPI and toe-brachial index (TBI) as a gold standard in the assessment of known or suspected PAD, in patients with and without severe aortic stenosis (AS), thus determining the feasibility of predicting peripheral arterial disease in this group.

## Material and methods

### Patient population

**Fig 1:**
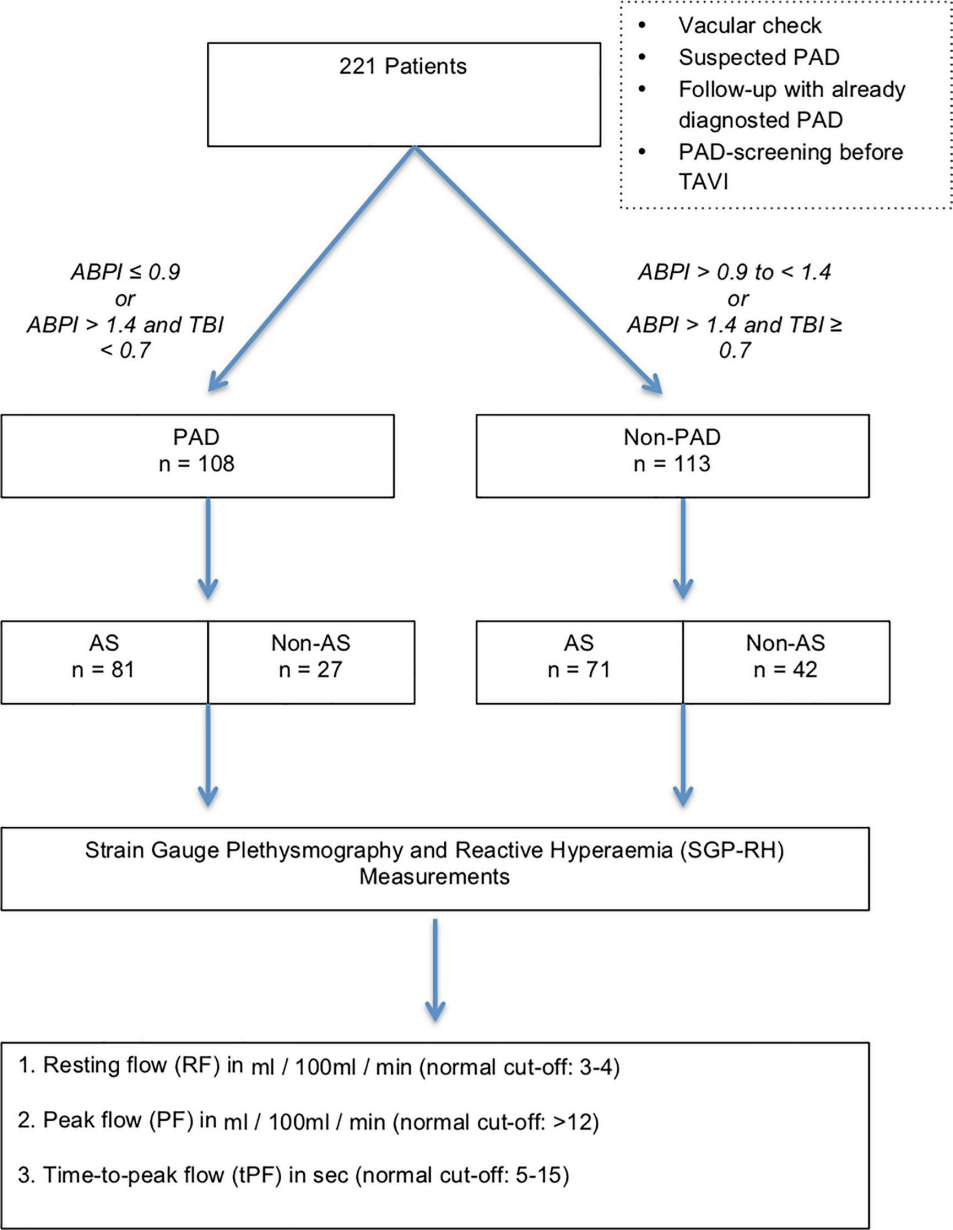
Study flow chart.

Between October 2015 and October 2017, a total of 221 patients were referred for a vascular check with suspected PAD, with already diagnosed PAD for follow-up, or for PAD screening in patients with AS before undergoing Transcatheter aortic valve implantation (TAVI) in the Department of Angiology. Data from these subjects were collected retrospectively and included into this single center study (Fig 1).

According to the standard care for PAD, study subjects underwent ABPI measurements in both legs (reference for presence of PAD ≤ 0.9). They underwent bilateral SGP-RH when they were not able to perform an exercise test, such as a treadmill test, for any reason, or depending on the physician’s decision. Ethics approval for this retrospective study was not necessary. Our local ethic committee of the faculty of medicine of the University of Bonn (Germany) confirmed that ethical approval was not needed in this case. Patients records used in our retrospective study were all fully anonymized before we accessed them and anlayzed it.

### Assessment of ABPI

The assessment of the ABPI is a simple, non-invasive clinical test to diagnose PAD.

ABPI was performed by measuring the systolic blood pressure from both brachial arteries and from the posterior- (PTA) and anterior-tibial (ATA) or the dorsalis pedis arteries. All measurements were performed with the use of appropriately sized pneumatic cuffs for both the ankle and the arm. The systolic ankle pressures were recorded with a handheld 5 MHz bi-directional pocket Doppler instrument by continuous wave (cw) velocity detection (Bidop ES-100V3, HADECO, Kawasaki, Japan). The ABPI was calculated for each lower limb as the ratio of the lowest pressure from the right or the left PTA and ATA (or dorsalis pedis) over the greatest brachial systolic pressure according to the following formula:

$$\mathrm{ABPI}=\frac{\mathrm{Lowest}\;\mathrm{systolic}\;\mathrm{pressure}\;\mathrm{from}\;\mathrm{the}\;\mathrm{right}\;\mathrm{or}\;\mathrm{the}\;\mathrm{left}\;\mathrm{PTA}\;\mathrm{and}\;\mathrm{ATA}}{\mathrm{Greatest}\;\mathrm{systolic}\;\mathrm{pressure}\;\mathrm{from}\;\mathrm{the}\;\mathrm{right}\;\mathrm{or}\;\mathrm{left}\;\mathrm{brachial}\;\mathrm{artery}}$$

For each patient, only the lowest ABPI recorded in the two ankles was used for categorization into non-PAD and PAD groups. Ratios of > 0.90 to < 1.4 are considered normal, and ratios ≤ 0.9 indicate the presence of PAD [2,7,8,9]. For the present study, PAD severity is based on the following ABPI values: ABPI 0.75–0.90 = mild PAD; ABPI 0.50–0.75 = moderate PAD; ABPI < 0.50 = severe PAD.

In patients with an ABPI ≥ 1.4, which were considered to have medial calcific sclerosis with non-compressible arteries, the criteria for the classification into the PAD or non-PAD group were determined by additional toe-pressure measurements (TBI, see below).

### Assessment of TBI

In many cases, TBI is useful in medical conditions associated with medial calcifications (called Mönckeberg’s sclerosis), such as advanced age, longstanding diabetes, or chronic kidney disease, which can lead to unreliable ABPIs due to non-compressible vessels [10]. However, because the vessels in the toe are less susceptible to vessel stiffness [11], TBI is a better way of assessing PAD in these patients.

The TBI was determined as the ratio of the pressure from the right and the left big toe, measured by means of a photoplethysmograph (PPG) infrared light sensor (vasolab®320, ELCAT GmbH, Wolfratshausen, Germany), over the greatest brachial systolic pressure measured. Ratios less than 0.70 are considered appropriate to define PAD [2].

$$\mathrm{TBI}=\frac{\mathrm{Systolic}\;\mathrm{pressure}\;\mathrm{from}\;\mathrm{the}\;\mathrm{right}\;\mathrm{or}\;\mathrm{the}\;\mathrm{left}\;\mathrm{big}\;\mathrm{toe}}{\mathrm{Greatest}\;\mathrm{systolic}\;\mathrm{pressure}\;\mathrm{from}\;\mathrm{the}\;\mathrm{right}\;\mathrm{or}\;\mathrm{the}\;\mathrm{left}\;\mathrm{brachial}\;\mathrm{artery}}$$

### Assessment of SGP-RH

The assessment of SGP-RH is a simple method for measuring peripheral blood flow that has been in use for more than 100 years and allows physicians to diagnose and further evaluate PAD. Despite its lengthy history, its current importance/prevalence for both clinical use and research is rather low and mostly limited to the assessment of venous diseases of the lower legs [12], because the method has proven to be prone to errors and artefacts that make the interpretation of the results difficult.

SGP-RH is based on the principle of post-occlusive reactive hyperaemia (RH), and offers the examiner the opportunity to register blood flow and volume changes after a certain period of hyperaemia by applying controlled pressure to a cuff around a segment of the limb. These measurements are, in turn, designed to provide information on the ability of the arterial inflow to respond to an ischemic stimulus [6] especially in patients who are unable to perform an exercise test (e.g. treadmill exercise) because of an underlying illness, such as severe symptomatic aortic stenosis or chronic lung disease. Finally, SGP-RH allows the physician to draw a conclusion as to the level of severity of the arterial perfusion disturbance and the level of compensation in the presence of PAD.

### Experimental setup

Before the peripheral blood flow measurements, the patients were invited to rest in a supine position for at least 15 minutes. The patient’s legs were positioned in such a way that the knee was neither flexed nor hyperextended while lying on the examination couch, and the lower legs were placed slightly above heart level (at an angle of approximately 30 degrees) by supporting the heels with the use of specially tailored foam cushions and pillows. Constant room temperature was maintained at an average of 20°C. Peripheral blood flow measurements were conducted in both legs simultaneously. For the testing, both appropriately sized pneumatic occlusion cuffs were wrapped around the patient’s thighs and the strain gauge detectors were circularly placed on the thickest part of the calf and connected to a plethysmography device (vasolab®320, ELCAT GmbH, Wolfratshausen, Germany).

This method enables the evaluation of the following parameters:

1. Resting flow (RF) in ml / 100ml / min (normal cut-off: 3–4)
2. Peak flow (PF) in ml / 100ml / min (normal cut-off: >12)
3. Time-to-peak flow (tPF) in sec (normal cut-off: 5–15)

The peripheral blood flow measurements consisted of two stages:

#### (1) Measurement of resting flow (RF)

For the measurement of the resting flow (RF), the pneumatic occlusion cuffs of the thigh were inflated up to 60 mmHg to guarantee venous occlusion. For the testing, three consecutive pressure inflations were performed (three times per minute) for 5 seconds each with a 15 second interval in between each occlusion in which the cuff was instantaneously deflated. As a result, RF values were obtained at 0, 15, and 30 seconds.

Normal RF values can reach 3–4 ml / 100ml / min. In patients with mild or moderate PAD (Fontaine’s stage II/III), the blood flow at rest remains within in the normal range, whereas a reduction of the RF becomes apparent in patients with severe PAD and pain or discomfort while resting [13].

#### (2) Measurement of reactive hyperaemia (RH)

For the measurement of reactive hyperaemia (RH), the pneumatic occlusion cuffs on the thigh were inflated to a suprasystolic pressure to exclude peripheral circulation (standard: 180 mmHg, in hypertensive patients: 30–40 mmHg above systolic blood pressure). After three minutes, the arterial occlusion was released. Hereafter, five consecutive pressure inflations up to 60 mmHg (to guarantee venous occlusion) were performed for 5 seconds each, with a 15-second interval in between each occlusion in which the cuff was instantaneously deflated. As a result, RH values were obtained at 5, 15, 25, 35, and 45 seconds.

After a three-minute period of interruption of blood flow, the normal arterial blood inflow may reach 20–40 ml / 100ml / min in healthy adults. This value correlates with the severity of the disease and decreases steadily with progression through the stages of PAD: In patients with Fontaine’s stage II PAD, the arterial blood inflow ranges between 4–10 ml / 100ml / min, whereas in patients with advanced stage PAD (Fontaine’s stage III or IV), the arterial blood inflow is considerably reduced (1–5 ml / 100ml / min) [13]. The cut-off for pathological arterial blood inflow (PF) in our study was less than 12 ml / 100ml / min.

A subsequent computer-assisted analysis factored in first flow, peak flow (PF), and time-to-peak flow (tPF). The first flow was defined as the first arterial blood inflow during hypaeremia after three minutes of circulatory arrest (in ml / 100ml / min). The PF was recorded as the maximal arterial inflow during hypaeremia after three minutes of circulatory arrest (in ml / 100ml / min). The tPF was specified as the time (in seconds) until the peak flow had been reached.

The PF and the tPF are normal if the greatest arterial inflow occurs following the first occlusion maneuver and constantly decreases during subsequent maneuvers. If the arterial inflow behaves differently, then the PF is pathological. The same applies to the tPF; a healthy, no-PAD patient will have reached the maximum within 5 to 15 seconds. Any discrepancies thereof (peak flow happens with delay or not at all) are considered pathological. In the present study, the cut-off for pathological tPF was >15 seconds. In conclusion, a PF greater than 12 ml/ 100ml / min and a tPF within 15 seconds with a fast decrease afterwards indicates a good peripheral arterial perfusion reserve. Deviations from this indicate the presence of PAD [13, 14].

### Statistical analysis

Data for continuous variables are given as the mean ± standard deviation. Categorical variables are given as frequencies and percentages. For comparisons between two groups, a Student’s t-test was performed for continuous variables and a Chi-square test for categorical data; the Chi-square test was also used for categorical variables. Sensitivity and specificity were calculated with 95% confidence limits. ROC curves for PF and tPF were plotted to show the predictive potential of these variables with respect to PAD. Statistical analyses were conducted with SPSS Statistics Version 22.0 (IBM Corporation, Somer, NY).

The investigators initiated the study, had full access to the data, and wrote the manuscript. All authors vouch for the accuracy and completeness of the data and analyses and confirm that the study was conducted according to the protocol.

## Results

### Baseline characteristics

**Fig 2:**
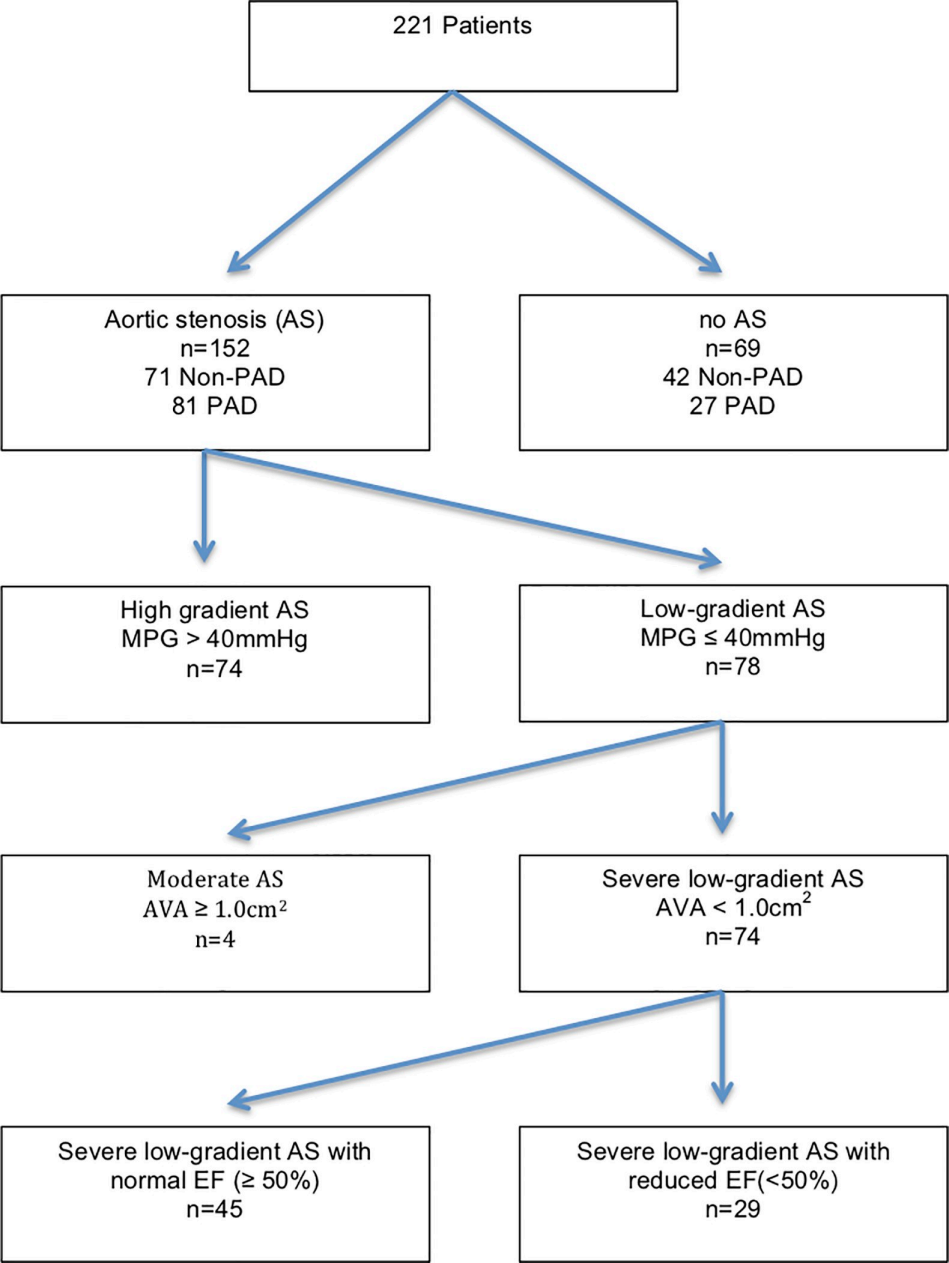
Flow chart for patients with Aortic stenosis according to flow-gradient patterns.

Patients’ baseline characteristics, according to vascular status, are summarized in Table 1. 221 patients (133 men, mean age: 77 ± 10.6 years, mean BMI: 26.5 ± 4.3) were assessed for PAD. Patients were subdivided into two main groups according to the presence or absence of PAD, which was defined with reference to the ABPI and TBI measurements (Fig 1). 48.9% (n = 108) of our patients suffered from PAD. 41.7% of them had medial sclerosis, 32.4% mild, 19.4% moderate, and 6.5% severe PAD. There were 68.7% (n = 152) patients with AS and 69 non- AS. 74 Patients in AS group had a high gradient AS and 78 low-gradient AS (Fig 2). The sensitivity of flow measurements was analyzed separately for this group. PAD patients suffered significantly more from Diabetes mellitus (PAD n = 41 (37.9%) vs. non-PAD n = 25 (22.1%); p = 0.004). 60.1% of PAD patients had coronary artery disease, 47.2% had carotid artery disease (Table 1).

**Table 1:** Baseline characteristics for non-PAD and PAD patients.

	All patients (n = 221)	Non-PAD^a^ (n = 113 (51,1%))	PAD (n = 108 (48,9%))	p-value
Age (years)	77.4 ± 10.6	75.9 ± 11.5	78.9 ± 9.2	0.032
Male gender, n (%)	133 (60.1)	73 (64.6)	60 (55.56)	0.042
Arterial Hypertension (%)	122 (55.2)	61 (53.9)	61 (56.48)	0.100
Body mass index (kg/m^2^)	26.5 ± 4.3	25.9 ± 3.4	27.1 ± 5.0	0.041
Diabetes mellitus, n (%)	66 (29.8)	25 (22.1)	41 (37.9)	0.004
CAD^b^ n (%)	122 (55.2)	57 (50.4)	65 (60.1)	0.037
Previous MI^c^, n (%)	32 (14.4)	17 (15.0)	15 (13.8)	0.147
Carotid disease, n (%)	87 (39.3)	36 (31.8)	51 (47.2)	0.007
Previous stroke, n (%)	22 (9.9)	7 (6.1)	15 (13.8)	0.029
COPD^d^, n (%)	20 (9.0)	11 (9.7)	9 (8.3)	0.173
**Severe Aortic stenosis, n (%)**	**152 (68.7)**	**71 (62.8)**	**81 (75.0)**	**0.017**
**PAD disease classification**		(n = 108)		
Medial calcific sclerosis, n (%) (ABPI > 1.4 and TBI < 0.7)			45 (41.7)	
Mild PAD, n (%) (ABPI 0.75–0.90)			35 (32.4)	
Moderate-severe PAD, n (%) (ABPI 0.50–0.75)			21 (19.4)	
Severe PAD, n (%) (ABPI < 0.50)			7 (6.5)	

^a^peripheral arterial disease

^b^coronary artery disease

^c^myocardial infarction

^d^chronic obstructive pulmonary disease

Values are given as frequencies and percentages, mean ± SD

#### (1) Assessment of resting flow (RF)

**Fig 3:**
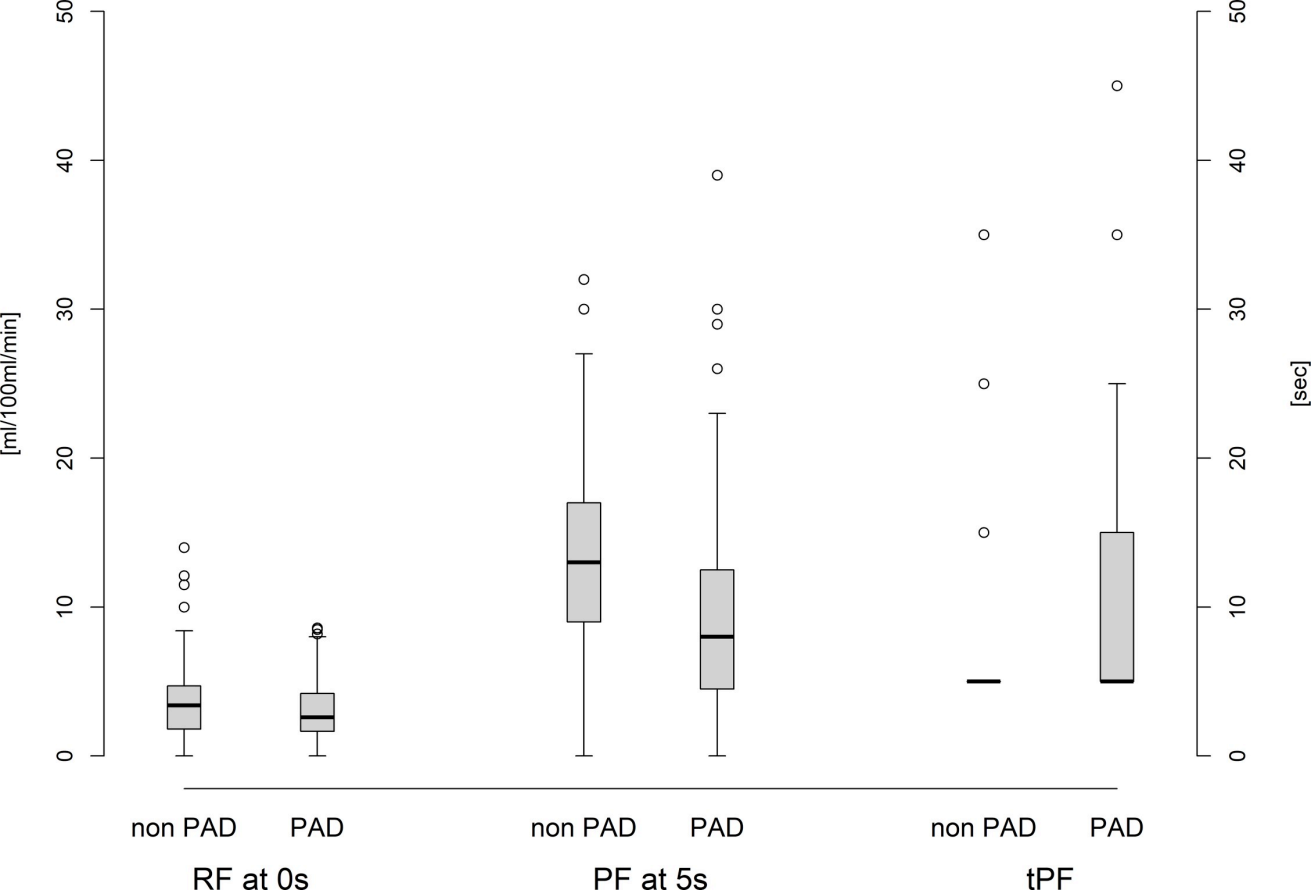
Resting Flow (RF), Peak Flow (PF), and Time-to-peak flow (tPF) in non-PAD and PAD patients.

Irrespective of AS in patients with and without PAD, the resting arterial blood flow showed the highest level after 0 seconds and constantly decreased afterwards. At 0 seconds, the RF was within the normal range and reached 3–4 ml / 100ml / min, irrespective of whether PAD was present or not, however PAD patients showed lower values than non-PAD patients (Table 2, Fig 3).

**Table 2:** Resting flow and reactive hyperaemia values (PF and tPF) in non-PAD and PAD patients.

	Non-PAD (n = 113)	PAD (n = 108)	p-value
Resting flow (RF) ml / 100ml / min			
at 0 sec^a^	3.6±2.51	3.1±2.09^b^	0.123
at 15 sec	3.01±1.90	2.69±1.65	0.188
at 30 sec	3.03±1.86	2.70 ±1.72	0.172
Reactive hyperemia:			
Peak flow (PF) (ml / 100ml / min)			
** at 5 sec**	**13.51±6.46**	**9.17±6.93**^**b**^	**<0.0001**
at 15 sec	8.32±4.37	6.68±4.75	0.008
at 25 sec	6.34±3.88	5.34±3.89	0.058
at 35 sec	5.08±3.42	4.69±3.35	0.385
at 45 sec	4.99±3.41	4.36±3.31	0.165
**Time-to-peak flow in sec (tPF)**	**6.15±4.17**^**c**^	**11.75±9.84**^**c**^	**<0.0001**

^a^Irrespective of aortic Stenosis (AS) in patients with and without peripheral arterial disease (PAD), the resting arterial blood flow showed the highest level after 0 seconds.

^b^PAD patients showed lower Resting Flow (RF) and Peak Flow (PF) than non-PAD patients.

^c^In patients with PAD and without PAD, the time-to-peak flow (tPF) was within the normal range, but significantly prolonged in PAD patients

#### (2) Assessment of reactive hyperaemia

(2.1) Assessment of peak flow

**Fig 4:**
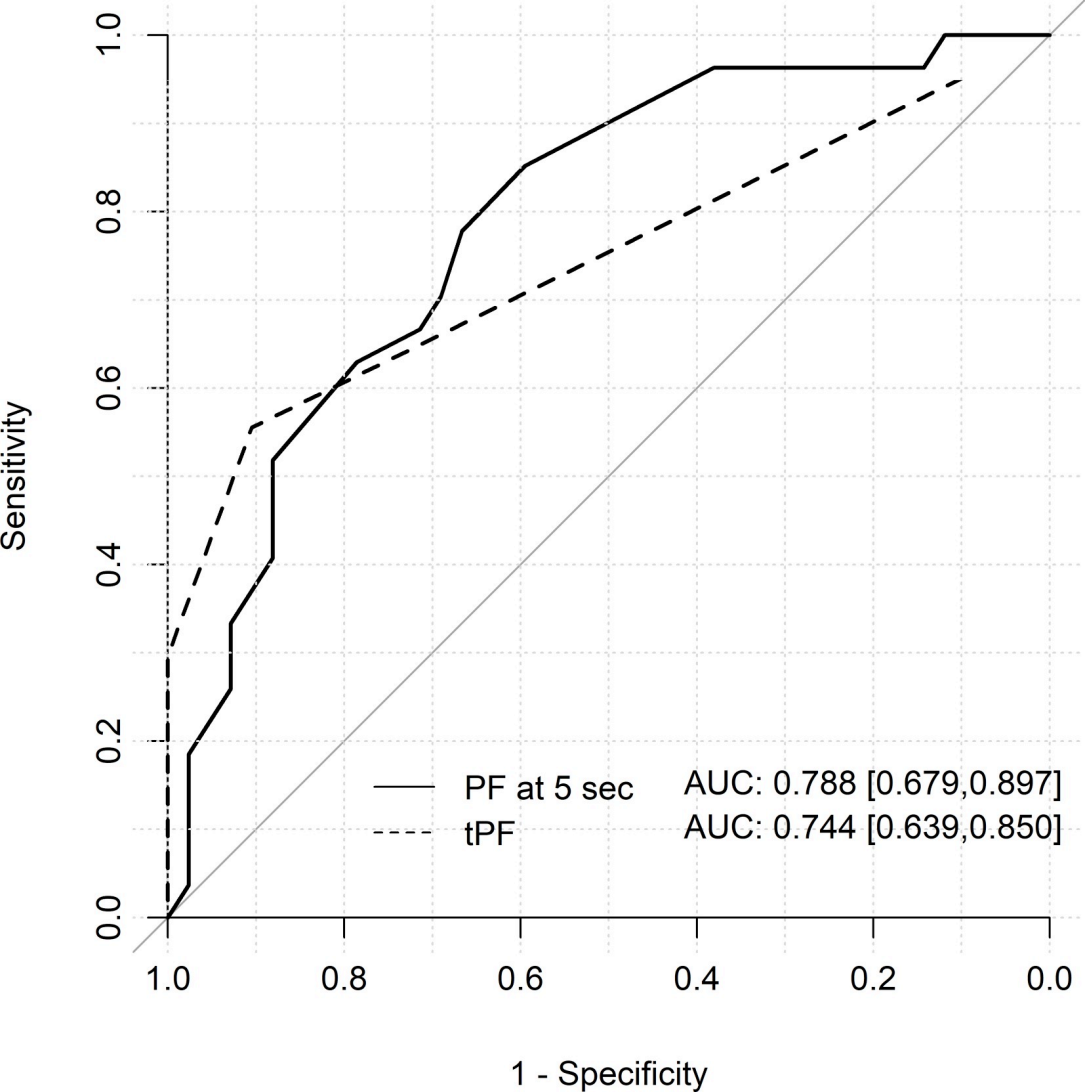
ROC curves for Peak Flow at 5 seconds and Time-to-peak flow in patients without severe aortic stenosis (AS). The curves show the predictive potential of PF at 5 seconds (solid line) and tPF (dashed line) variables with respect to PAD in patients without AS. PF at 5 seconds and tPF correlated better with ABPI in patients without AS.

**Fig 5:**
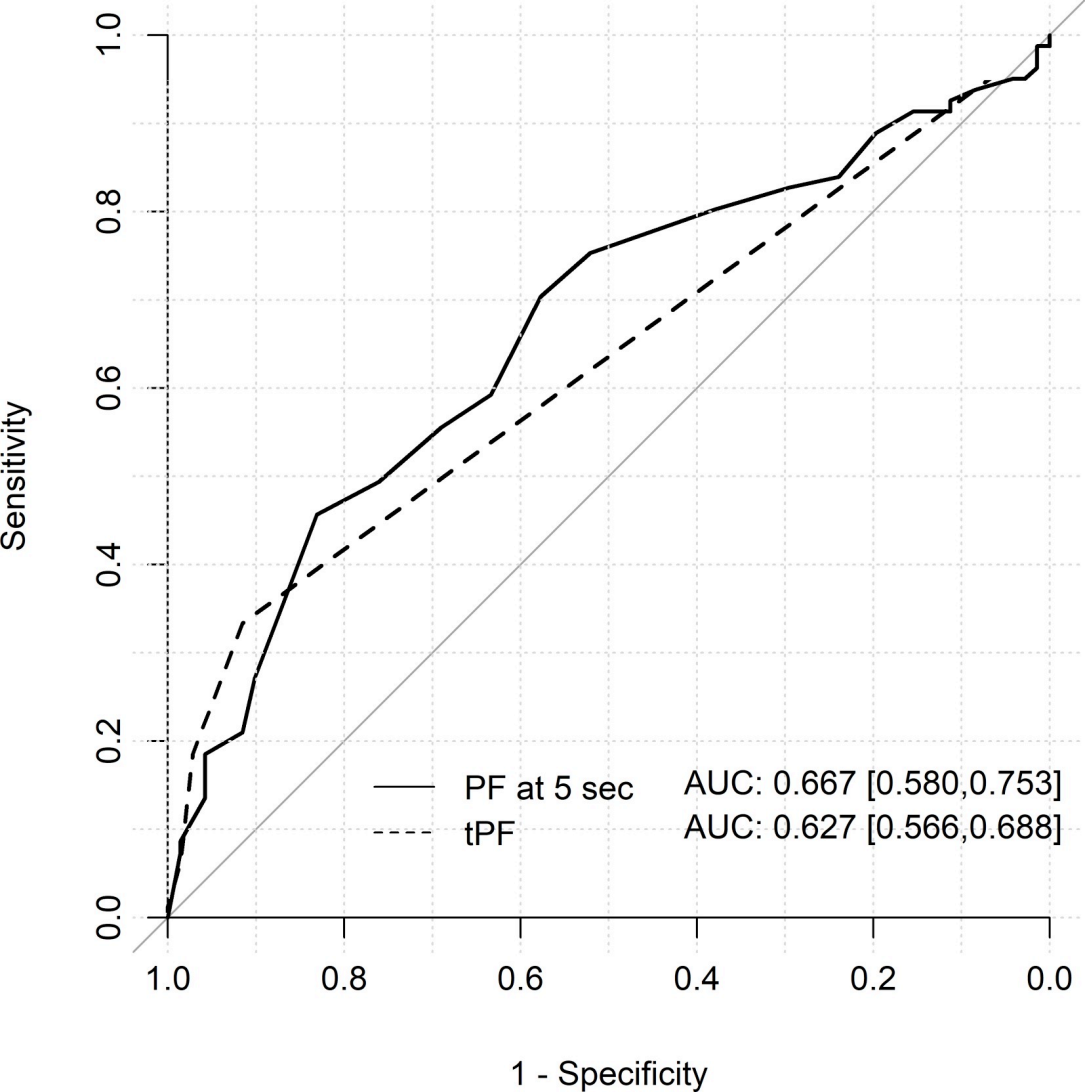
ROC curves for Peak Flow at 5 seconds and Time-to-peak flow in patients with severe aortic stenosis (AS). The curves show the predictive potential of PF at 5 seconds (solid line) and tPF (dashed line) variables with respect to PAD in patients with AS. PF at 5 seconds correlated well with ABPI; the tPF correlate less with ABPI in this group.

Regardless of whether PAD was present or not, the peak flow (PF) showed the highest level after 5 seconds and constantly decreased afterwards. PAD patients showed a significantly lower value (all results as follows: non-PAD vs. PAD 13.51±6.46 ml / 100ml / min vs. 9.17±6.93 ml / 100ml / min, p < 0.0001). In PAD patients, all PF values were below the standard level. In non-PAD patients, the PF at 5 seconds was within the normal range (Table 2, Fig 3). The PF at 5 seconds correlated well with ABPI and allowed us to detect PAD in patients with AS (AUC 0.667; CI 0.580–0.753) and without AS (AUC 0.788; CI 0.679–0.897; Figs 4 and 5).

(2.2) Assessment of time-to-peak flow

In patients with PAD and without PAD, the time-to-peak flow (tPF) was within the normal range (normal: 5–15 sec), but significantly prolonged in PAD patients (PAD vs. non-PAD; 11.75±9.87 sec vs 6.15±4.17 sec, p < 0.0001; Table 2, Fig 3). In patients with AS, the tPF correlated less with ABPI and couldn´t detect PAD and was a false negative. In patients without AS, tPF correlated well with ABPI (AUC 0.744; CI 0.639–0.850) and predicted PAD (Fig 4).

**Fig 6:**
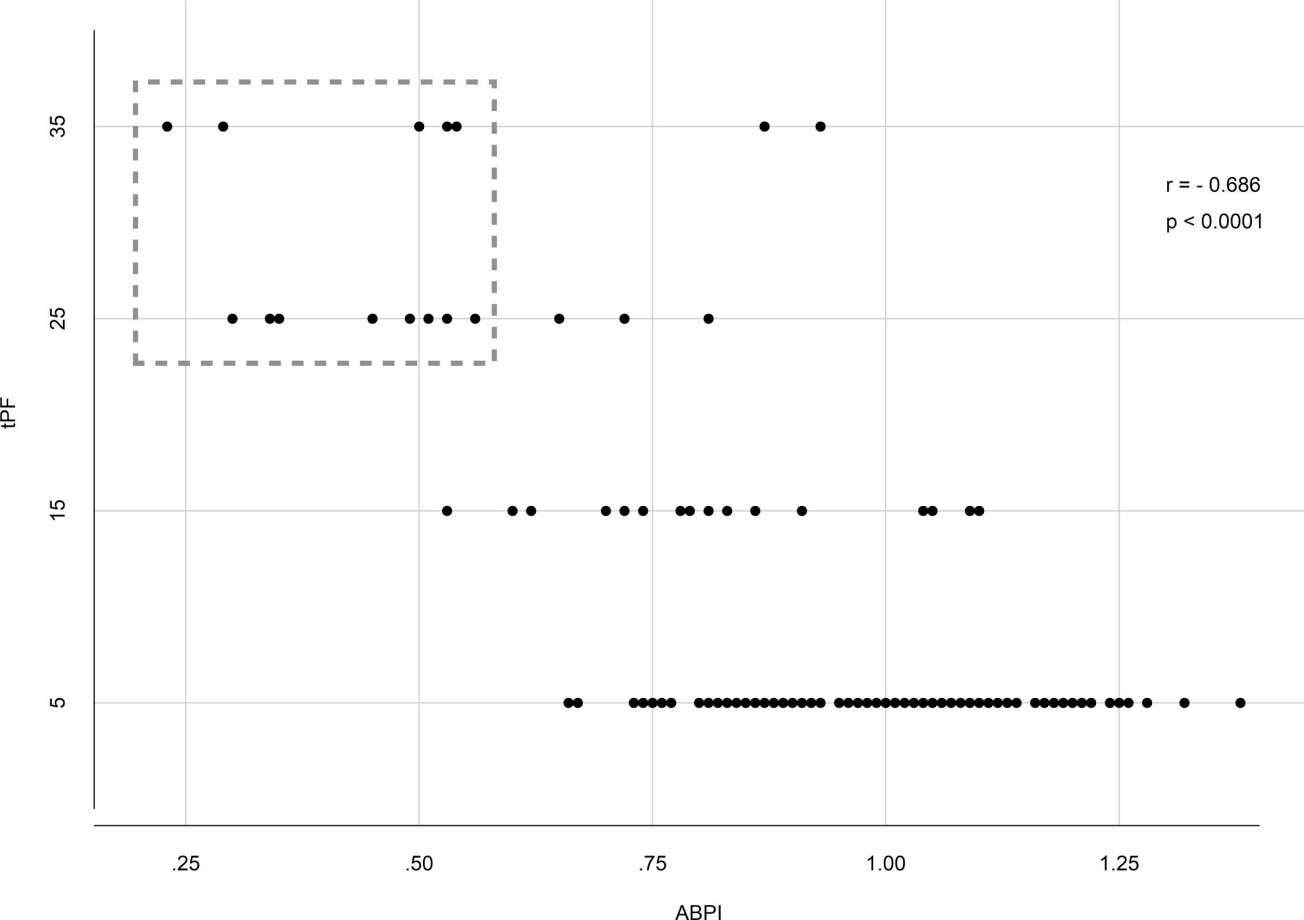
Scatter plot for the correlation between tPF (vertical axis) and ABPI (horizontal axis). There is a significant correlation between tPF in PAD patients with ABPI values less than 0.7 (dash-lined box in the plot).

On the other hand, there was a significant correlation between tPF and low ABPI. Especially patients with an ABPI ≤ 0.7 have significant pathological tPF values (Fig 6). Table 3 shows the accuracy of SGP-RH values in our collective. It shows that time to peak flow and resting flow at 0 sec had a specificity of 98% and 94% with a very low sensitivity and PF at 5sec had a sensitivity of 75% with a low specificity in this collective.

**Table 3:** Sensitivity, specificity, positiv predictive and negative predictive values of SGP-RH parameters (RF, PF at 5sec and tPF) in PAD patients.

	Sensitivity %	Specificity %	+ predictive %	- predicitve %
**ABPI ≤ 0.9 and TBI ≤ 0.7**				
Resting flow at 0 sec: Cut-off < 1 ml / 100ml / min	< 50	94	46	51
Peak flow at 5 sec: Cut-off < 12 ml / 100ml / min	75	55	61	70
Time to peak flow: Cut-off > 15 sec	<50	98	92	57

The diagnostic tests (RF, PF at 5sec and tPF) in patients with low-gradient AS didn´t correlate well with ABPI in detecting PAD (AUC : RF 0.582, PF at 5sec 0.484 and tPF 0.575).

However PF at 35sec and 45sec correlated better with ABPI (AUC 0.604 and 0.608) in patients with low flow low gradient stenosis, but not in Patients with high flow gradient.

**Fig 7:**
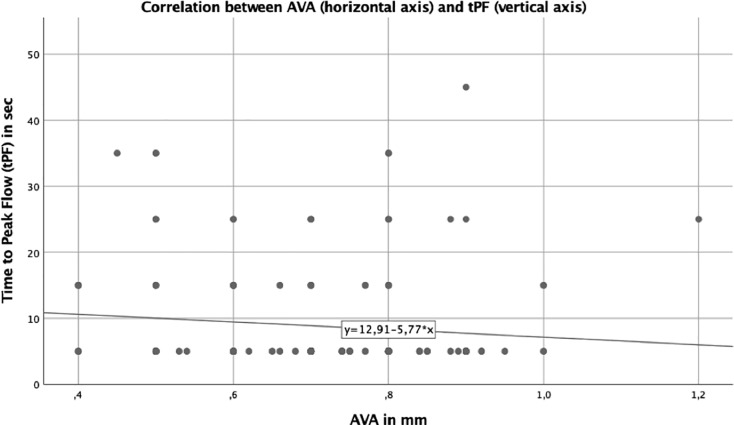
Correlation between AVA and tPF in patients with AS. The scatter plot shows a positive trend between AVA reduction and prolonged tPF in patients with AS.

In AS population aortic valve area (AVA) correlated parametically well with tPF (p = 0.042). The scatter plot (Fig 7) shows positive trend between reduced AVA and prolonged tPF. We didn´t find any correlation between Pmean and tPF.

## Discussion

### Resting flow

Irrespective of whether PAD was present or not, the RF at 0 seconds was within the normal range in both groups and reached 3–4 ml / 100ml / min. RF measurements appear to be a rather unspecific parameter, which did not provide any substantial information. These values are considered normal and are consistent with previous findings: research from Bossaert et al. [15] has established that the blood circulation of the lower extremities is rather low in contrast to the cerebral or renal perfusion under resting conditions; the human body makes use of merely 15 percent of the entire cardiac output to guarantee proper blood flow in both the upper and lower extremities. In healthy individuals, scintigraphic measurements with xenon radionuclides resulted in values around 3–4 ml of blood per minute per 100 ml of tissue (ml / min / 100 ml) [15]. In vascular patients, however, a reduction in resting flow would have been reasonably expected because of the narrowing of the arteries. The fact that RF values were in the normal range in the PAD group might be due to the fact that PAD patients can compensate for disturbed and/or impaired blood circulation in small vessels by reducing the peripheral vascular resistance or that our patients had mostly mild to moderate forms of PAD, as indicated in the baseline characteristics section. Measurement of peripheral blood flow at rest is unspecific, because there is an association between patient’s daily mood, external psychogenic stimuli, environmental conditions and flow values [16]. Furthermore, resting flow rates fail to make a distinction between normal subjects and patients with arterial disease having an adequate supply through the collateral circulation to cope with the basal requirements of the tissues [17].

Finally, at this time, it is not possible to give a reliable statement on the importance of RF in terms of the peripheral perfusion.

### Reactive hyperaemia

#### Peak flow

Our study shows that peak flow at 5 seconds was the only flow value, that correlated well with ABPI in detecting PAD in patients with (AUC 0.7; CI 0.580–0.753) and without AS (AUC 0.8; CI 0.679–0.897). Reactive hyperaemia is a reproducable method to assess the extent of artrial inflow initiated by an ischemic stimulus [18,19]. Normal subjects experience an increase of blood flow directly after cuff deflation, a rapid return to baseline levels is expected in normal extremities. Under physical stress an increase in muscle blood circulation by up to 80 ml / 100ml / min can be obtained by reactive hyperaemia as a physiological response to stress or following short-term ischemia [20]. In the presence of an obstruction or stenosis of the lower extremity vessels, there is a reduction of peak flow, and a delay of the time to reach maximal flow. Findings in our present case series of patients indicate an association between PAD and delay in reaching peak flow, hence a prolonged tPF and decreased maximal PF. In our study, we found flow values do not serve as an important predicters at the level and degree of the disease.

#### Time-to-peak flow

In our study population with and without AS, PAD was not detected in tPF values with a cut-off point of > 15 sec, but it was significantly prolonged in the PAD group, compared with non-PAD, and there was a significant correlation with the ABPI measurement (Fig 6). Fig 6 shows specifically that patients with ABPI < 0.7 had prolonged tPF values (> 15 sec). This group with moderate (19.4%) and severe (6.5%) stages of PAD were underrepresented with, in total, 25.9% of the study population [13, 14]. On the other hand, the cut-off point for tPF is based on a few older studies with small numbers of patients [13, 14], so we need larger, randomized, controlled studies with enough power to verify the significant cut-off-points for the value. Interestingly, in the subgroup without AS, tPF correlated well with ABPI (AUC 0.744; CI 0.639–0.850) in detecting PAD.

In subjects with PAD atherosclerotic changes and endothelial dysfunction can alter the strain-gauge measurements, as compared to healthy individuals [21]. A decrease of the peripheral artrial systolic blood pressure is a result of functional impairment of the peripheral circulation. If the degree of stenosis increases, you notice a fall in the peak flow and a prolonged total time. Moreover delayed onset of the PF may be an expression of arterial obstruction. Not all patients with PAD show these characteristics. In our cohort, in the patients with AS, the time-to-peak flow (tPF) didn’t predict PAD and was considered a false negative. This can make it difficult to differentiate a normal vessel from an unhealthy vessel when analyzing the discussed flow characteristics.

To the best of our knowledge, this is the first study to evaluate SGP-RH parameters in AS patients. Physiological changes in pressure and flow within the arteries, spanning from the ascending aorta to the anterior tibial artery, show that in presence of a “healthy” aortic valve and with increasing distance from the heart, the maximum pressure impulse increases slightly at the detriment of the flow pulse (14). In cases of pre-existing high-degree aortic valve stenosis with reduced cardiac output the respective values are markedly reduced and may become difficult to detect PAD. In vascular patients; however, a prolonged tPF would have been reasonably expected because of narrowing of the arteries. The fact that expectations were not met in AS group might be due to the fact that PAD-patients may compensate disturbed and/or impaired blood circulation in small vessels by reducing peripheral vascular resistance. However in Patients with AS and low flow low gradient stenosis the PF at 35 and 45sec corraletd well with ABPI in detecting PAD, but not the PF at 5sec. Reasons for this will amongst others originate from abnormal haemodynamic flow patterns in low flow low gradient stenosis, local regulatory processes in AS patients and reduced ejection fraction that were in fact not yet known. We can only speculate that the diagnostic method is insufficiently sensitive in this group.

On the other hand, pathological changes like atherosclerosis and endothelial disfunction are assumed to be mechanisms by which patients with PAD may result with lower peak-blood-flow values and delayed peak-blood-flow response [21]. These results suggest insufficient support of skeletal muscle due to the inadequate oxygen delivery. Further main difference is that patients suffering from PAD need a longer time to reestablish baseline blood-flow values. In our study population, the first PF values were significantly reduced in PAD patients and we had a significantly prolonged tPF compared with the non-PAD group (Fig 6).

The strain gauge has been utilized in validity and reliability testing since the late 1800s. However, this method has proven to be prone to errors and artefacts, making the interpretation of results difficult. Precise analysis is dependent upon accurate equipment set-up and limb positioning in order to prevent the over- or underestimation of peak blood flow while maximizing reliability. Salisbury et al. [22] emphasized that the strain-gauge method is a useful methode in evaluating the prognosis and assessing the efficacy of therapeutic interventions in patients with PAD, particularly following interventions such as a supervised exercise program. Therefore, to evaluate the effectiveness of disease management strategies, SGP-RH is considered a helpful analytical technique [22].

### Study limitations

This study was designed to examine the feasibility and accuracy of SGP-RH values compared with the Doppler ultrasound method, ABPI, in the assessment of known or suspected PAD, especially in patients with severe aortic stenosis in our vascular center. This was a single-center experience that started in 2016. As such, the clinical assessment of peripheral arterial disease using objective clinical vascular tests was limited to only a small number of patients, therefore, we cannot reasonably address this issue. Given the small sample size, the proportion of PAD patients were not well balanced in terms of PAD severity, the majority of the PAD patients had only mild to moderate forms of peripheral arterial disease. For further verification and generalization of our results, larger studies that include more patients with higher-stage PAD are required. In the case of SGP-RH, however, the artefacts observed, which made interpretation of the results difficult, could be due to patients that do not tolerate the suprasystolic pressure of the pneumatic occlusion cuffs for the thigh and the resulting pain-triggered contractions of the lower-leg musculature. Other causes may include poor fit of the strain-gauge detectors or movement of the patients during the measurements.

## Conclusion

The result of the study shows fair accuracy and agreement between parameters of SGP-RH peak flow at 5 seconds and ABPI for the detection of peripheral artery disease in the lower limbs in patients with and without AS. Time-to-peak flow values had a better predictive potential in detecting PAD in patients without AS. In patients with AS, it is less sensitive. There is a significant correlation between tPF in PAD patients with ABPI values less than 0.7

All in all, flow measurements have a limited predictive potential for the detection of PAD in patients with severe aortic stenosis than ABPI.

From the economic point of view and availability, the Doppler ultrasound may be the method of first choice for measurement, but when the results are in contrast to the clinical findings or patients are unable to perform an exercise treadmill test, referral to SGP-RH measurement might be considered. Especially in patients with low ABPIs, the Doppler method often failed, most frequently at the dorsal pedal artery. Furthermore, SGP-RH measurement is a useful methode in evaluating the prognosis and assessing the efficacy of therapeutic interventions in patients with PAD.

## Supporting information

S1 Table Statistical analysis 1. (PDF)

S2 Table Statistical analysis 2. (PDF)
