# Supplementary material for: Comparative study of pressure (ankle-brachial pressure index) and flow (strain gauge plethysmography and reactive hyperaemia) measurements in diagnosis of peripheral arterial disease in patients with severe aortic stenosis
Source: PLoS One. 2019 Jul 30;14(7):e0220510. doi: 10.1371/journal.pone.0220510 (PMC6667209; doi:10.1371/journal.pone.0220510)
Supplement: S1 Table — (PDF) [file pone.0220510.s001.pdf]

pad	N	Mittelwert	Std.abw.	Std Err	Minimum	Maximum
0	113	75.9204	11.5597	1.0874	22.0000	91.0000
1	108	78.9537	9.2144	0.8867	37.0000	96.0000
Diff (1-2)		-3.0333	10.4796	1.4102		

pad	Methode	Mittelwert	95% CL Mean		Std.abw.	95% CL Std Dev	
0		75.9204	73.7657	78.0750	11.5597	10.2239	13.3003
1		78.9537	77.1960	80.7114	9.2144	8.1279	10.6387
Diff (1-2)	Gepoolt	-3.0333	-5.8127	-0.2540	10.4796	9.5833	11.5623
Diff (1-2)	Satterthwaite	-3.0333	-5.7992	-0.2675			

Methode	Varianzen	DF	t-Wert	Pr > |t|
Gepoolt	Gleich	219	-2.15	0.0326
Satterthwaite	Ungleich	212.23	-2.16	0.0317

Gleichheit der Varianzen				
Methode	Num DF	Den DF	F-Wert	Pr > F
Folded F	112	107	1.57	0.0187

Variable: BMI (BMI)

pad	N	Mittelwert	Std.abw.	Std Err	Minimum	Maximum
0	113	25.9241	3.4722	0.3266	20.4500	39.7700
1	108	27.1176	5.0585	0.4867	17.6500	50.5000
Diff (1-2)		-1.1935	4.3206	0.5814		

pad	Methode	Mittelwert	95% CL Mean		Std.abw.	95% CL Std Dev	
0		25.9241	25.2769	26.5713	3.4722	3.0709	3.9950
1		27.1176	26.1527	28.0825	5.0585	4.4620	5.8404
Diff (1-2)	Gepoolt	-1.1935	-2.3394	-0.0476	4.3206	3.9511	4.7670
Diff (1-2)	Satterthwaite	-1.1935	-2.3499	-0.0372			

Methode	Varianzen	DF	t-Wert	Pr > |t|
Gepoolt	Gleich	219	-2.05	0.0413
Satterthwaite	Ungleich	188.54	-2.04	0.0431

Gleichheit der Varianzen				
Methode	Num DF	Den DF	F-Wert	Pr > F
Folded F	107	112	2.12	<.0001

Variable: LVEF (LVEF)

pad	N	Mittelwert	Std.abw.	Std Err	Minimum	Maximum
0	91	55.3626	12.0633	1.2646	25.0000	78.0000
1	88	55.6818	13.0530	1.3915	17.0000	79.0000
Diff (1-2)		-0.3192	12.5595	1.8777		

pad	Methode	Mittelwert	95% CL Mean		Std.abw.	95% CL Std Dev	
0		55.3626	52.8503	57.8749	12.0633	10.5292	14.1247
1		55.6818	52.9162	58.4475	13.0530	11.3684	15.3282
Diff (1-2)	Gepoolt	-0.3192	-4.0248	3.3865	12.5595	11.3763	14.0195
Diff (1-2)	Satterthwaite	-0.3192	-4.0301	3.3917			

Methode	Varianzen	DF	t-Wert	Pr > |t|
Gepoolt	Gleich	177	-0.17	0.8652
Satterthwaite	Ungleich	174.8	-0.17	0.8654

Gleichheit der Varianzen				
Methode	Num DF	Den DF	F-Wert	Pr > F
Folded F	87	90	1.17	0.4589

Variable: RR (RR)

pad	N	Mittelwert	Std.abw.	Std Err	Minimum	Maximum
0	91	144.0	26.0252	2.7282	90.0000	219.0
1	88	132.5	28.5206	3.0403	80.0000	250.0
Diff (1-2)		11.5447	27.2803	4.0786		

pad	Methode	Mittelwert	95% CL Mean		Std.abw.	95% CL Std Dev	
0		144.0	138.6	149.4	26.0252	22.7156	30.4726
1		132.5	126.4	138.5	28.5206	24.8399	33.4919
Diff (1-2)	Gepoolt	11.5447	3.4957	19.5937	27.2803	24.7103	30.4516
Diff (1-2)	Satterthwaite	11.5447	3.4825	19.6070			

Methode	Varianzen	DF	t-Wert	Pr > |t|
Gepoolt	Gleich	177	2.83	0.0052
Satterthwaite	Ungleich	174.28	2.83	0.0053

Gleichheit der Varianzen				
Methode	Num DF	Den DF	F-Wert	Pr > F
Folded F	87	90	1.20	0.3899

pad	N Beob	Variable	Etikett	N	Mittelwert	Std.abw.

0	113	Arterielle_Ruheeinstrom_Indexbei<br>Arterielle_Ruheeinstrom_Indexbe1<br>Arterielle_Ruheeinstrom_Indexbe2<br>Arterielle_Reserve_Indexbein_pre<br>Arterielle_Reserve_Indexbein_pr1<br>Arterielle_Reserve_Indexbein_pr2<br>Arterielle_Reserve_Indexbein_pr3<br>Arterielle_Reserve_Indexbein_pr4<br>Peak_Flow_Indexbein_pre	Arterielle_Ruheeinstrom_Indexbein_pre_0s<br>Arterielle_Ruheeinstrom_Indexbein_pre_15s<br>Arterielle_Ruheeinstrom_Indexbein_pre_30s<br>Arterielle_Reserve_Indexbein_pre_5s<br>Arterielle_Reserve_Indexbein_pre_15s<br>Arterielle_Reserve_Indexbein_pre_25s<br>Arterielle_Reserve_Indexbein_pre_35s<br>Arterielle_Reserve_Indexbein_pre_45s<br>Peak_Flow_Indexbein_pre	113<br>113<br>113<br>113<br>113<br>112<br>112<br>113<br>113	3.6017699<br>3.0168142<br>3.0380531<br>13.5132743<br>8.3274336<br>6.3482143<br>5.0892857<br>4.9911504<br>6.1504425	2.5139604<br>1.9063289<br>1.8617541<br>6.4657270<br>4.3761580<br>3.8897181<br>3.4289535<br>3.4186870<br>4.1730882
1	108	Arterielle_Ruheeinstrom_Indexbei<br>Arterielle_Ruheeinstrom_Indexbe1<br>Arterielle_Ruheeinstrom_Indexbe2<br>Arterielle_Reserve_Indexbein_pre<br>Arterielle_Reserve_Indexbein_pr1<br>Arterielle_Reserve_Indexbein_pr2<br>Arterielle_Reserve_Indexbein_pr3<br>Arterielle_Reserve_Indexbein_pr4<br>Peak_Flow_Indexbein_pre	Arterielle_Ruheeinstrom_Indexbein_pre_0s<br>Arterielle_Ruheeinstrom_Indexbein_pre_15s<br>Arterielle_Ruheeinstrom_Indexbein_pre_30s<br>Arterielle_Reserve_Indexbein_pre_5s<br>Arterielle_Reserve_Indexbein_pre_15s<br>Arterielle_Reserve_Indexbein_pre_25s<br>Arterielle_Reserve_Indexbein_pre_35s<br>Arterielle_Reserve_Indexbein_pre_45s<br>Peak_Flow_Indexbein_pre	108<br>108<br>108<br>108<br>108<br>107<br>107<br>108<br>108	3.1194444<br>2.6990741<br>2.7074074<br>9.1750000<br>6.6842593<br>5.3457944<br>4.6906542<br>4.3601852<br>11.7592593	2.0955430<br>1.6585939<br>1.7218607<br>6.9398635<br>4.7522186<br>3.8950932<br>3.3556073<br>3.3118293<br>9.8425648

pad	*N Beob*	*Variable*	*Minimum*	*25. Perzentil*	*Median*	*75. Perzentil*	*Maximum*

0	113	Arterielle_Ruheeinstrom_Indexbei<br>Arterielle_Ruheeinstrom_Indexbe1<br>Arterielle_Ruheeinstrom_Indexbe2<br>Arterielle_Reserve_Indexbein_pre<br>Arterielle_Reserve_Indexbein_pr1<br>Arterielle_Reserve_Indexbein_pr2<br>Arterielle_Reserve_Indexbein_pr3<br>Arterielle_Reserve_Indexbein_pr4<br>Peak_Flow_Indexbein_pre	0<br>0.1000000<br>0<br>0<br>0<br>1.0000000<br>0<br>0<br>5.0000000	1.8000000<br>1.7000000<br>1.9000000<br>9.0000000<br>5.0000000<br>3.0000000<br>3.0000000<br>3.0000000<br>5.0000000	3.4000000<br>2.8000000<br>2.8000000<br>13.0000000<br>8.0000000<br>5.0000000<br>5.0000000<br>4.0000000<br>5.0000000	4.7000000<br>3.9000000<br>3.9000000<br>17.0000000<br>11.0000000<br>9.0000000<br>6.0000000<br>7.0000000<br>5.0000000	14.0000000<br>10.5000000<br>10.0000000<br>32.0000000<br>23.0000000<br>20.0000000<br>19.0000000<br>19.0000000<br>35.0000000
1	108	Arterielle_Ruheeinstrom_Indexbei<br>Arterielle_Ruheeinstrom_Indexbe1<br>Arterielle_Ruheeinstrom_Indexbe2<br>Arterielle_Reserve_Indexbein_pre<br>Arterielle_Reserve_Indexbein_pr1<br>Arterielle_Reserve_Indexbein_pr2<br>Arterielle_Reserve_Indexbein_pr3<br>Arterielle_Reserve_Indexbein_pr4<br>Peak_Flow_Indexbein_pre	0<br>0<br>0<br>0<br>0<br>0<br>0<br>0<br>5.0000000	1.6500000<br>1.3000000<br>1.4500000<br>4.5000000<br>3.0000000<br>2.0000000<br>2.0000000<br>2.0000000<br>5.0000000	2.6000000<br>2.3000000<br>2.7500000<br>8.0000000<br>6.0000000<br>4.0000000<br>4.0000000<br>4.0000000<br>5.0000000	4.2000000<br>3.9000000<br>3.9000000<br>12.5000000<br>10.0000000<br>9.0000000<br>7.0000000<br>6.0000000<br>15.0000000	8.6000000<br>7.0000000<br>8.0000000<br>39.0000000<br>27.0000000<br>16.0000000<br>14.0000000<br>17.0000000<br>45.0000000

Table of pad by Geschlecht

pad	*Geschlecht(Geschlecht)*		
Häufigkeit<br>Prozent<br>Prozent Zeile<br>Prozent Spalte	*0*	*1*	*Summe*

0	40<br>18.10<br>35.40<br>45.45	73<br>33.03<br>64.60<br>54.89	113<br>51.13
1	48<br>21.72<br>44.44<br>54.55	60<br>27.15<br>55.56<br>45.11	108<br>48.87
Summe	88<br>39.82	133<br>60.18	221<br>100.00

Statistiken für Tabelle von pad nach Geschlecht

Statistik	*DF*	*Wert*	*Prob*
Chi-Quadrat	1	1.8858	0.1697
Likelihood-Ratio Chi-Quadrat	1	1.8879	0.1694
Kontinuitätskorr. Chi-Quad.	1	1.5272	0.2165
Mantel-Haenszel Chi-Quadrat	1	1.8773	0.1706
Phi-Koeffizient		-0.0924	
Kontingenzkoeffizient		0.0920	
Cramers V		-0.0924	

Exakter Test von Fisher	
Zelle (1,1) Häufigkeit (F)	40
Linksseitige Pr <= F	0.1082
Rechtsseitige Pr >= F	0.9346
Tabellenwahrscheinlichkeit (P)	0.0429
Zweiseitige Pr <= P	0.2160

Odds Ratio und relative Risiken			
Statistik	*Wert*	*95% Confidence Limits*	
Odds-Ratio	0.6849	0.3987	1.1766
Relatives Risiko (Spalte 1)	0.7965	0.5747	1.1038
Relatives Risiko (Spalte 2)	1.1628	0.9360	1.4446

Stichprobengröße = 221

Table of pad by Art_Hypertonie	
pad	*Art_Hypertonie(Art_Hypertonie)*

Häufigkeit<br>Prozent<br>Prozent Zeile<br>Prozent Spalte	0	1	Summe
0	52<br>23.53<br>46.02<br>52.53	61<br>27.60<br>53.98<br>50.00	113<br>51.13
1	47<br>21.27<br>43.52<br>47.47	61<br>27.60<br>56.48<br>50.00	108<br>48.87
Summe	99<br>44.80	122<br>55.20	221<br>100.00

Statistiken für Tabelle von pad nach Art_Hypertonie

Statistik	DF	Wert	Prob
Chi-Quadrat	1	0.1395	0.7088
Likelihood-Ratio Chi-Quadrat	1	0.1395	0.7088
Kontinuitätskorr. Chi-Quad.	1	0.0567	0.8118
Mantel-Haenszel Chi-Quadrat	1	0.1388	0.7094
Phi-Koeffizient		0.0251	
Kontingenzkoeffizient		0.0251	
Cramers V		0.0251	

Exakter Test von Fisher	
Zelle (1,1) Häufigkeit (F)	52
Linksseitige Pr <= F	0.6945
Rechtsseitige Pr >= F	0.4060
Tabellenwahrscheinlichkeit (P)	0.1004
Zweiseitige Pr <= P	0.7869

Odds Ratio und relative Risiken			
Statistik	Wert	95% Confidence Limits	
Odds-Ratio	1.1064	0.6508	1.8808
Relatives Risiko (Spalte 1)	1.0574	0.7886	1.4179
Relatives Risiko (Spalte 2)	0.9558	0.7537	1.2119

Stichprobengröße = 221

Table of pad by Diabetes_mellitus			
pad	Diabetes_mellitus(Diabetes_mellitus)		
Häufigkeit<br>Prozent<br>Prozent Zeile<br>Prozent Spalte	0	1	Summe
0	88<br>39.82<br>77.88<br>56.77	25<br>11.31<br>22.12<br>37.88	113<br>51.13
1	67<br>30.32<br>62.04<br>43.23	41<br>18.55<br>37.96<br>62.12	108<br>48.87
Summe	155<br>70.14	66<br>29.86	221<br>100.00

Statistiken für Tabelle von pad nach Diabetes_mellitus

Statistik	DF	Wert	Prob
Chi-Quadrat	1	6.6142	0.0101
Likelihood-Ratio Chi-Quadrat	1	6.6585	0.0099
Kontinuitätskorr. Chi-Quad.	1	5.8796	0.0153
Mantel-Haenszel Chi-Quadrat	1	6.5843	0.0103
Phi-Koeffizient		0.1730	
Kontingenzkoeffizient		0.1705	
Cramers V		0.1730	

Exakter Test von Fisher	
Zelle (1,1) Häufigkeit (F)	88
Linksseitige Pr <= F	0.9968
Rechtsseitige Pr >= F	0.0075
Tabellenwahrscheinlichkeit (P)	0.0043
Zweiseitige Pr <= P	0.0124

Odds Ratio und relative Risiken			
Statistik	Wert	95% Confidence Limits	
Odds-Ratio	2.1540	1.1938	3.8866
Relatives Risiko (Spalte 1)	1.2553	1.0514	1.4988
Relatives Risiko (Spalte 2)	0.5828	0.3823	0.8884

Stichprobengröße = 221

Table of pad by PAVK			
pad	*PAVK(PAVK)*		
Häufigkeit<br>*Prozent*<br>*Prozent Zeile*<br>*Prozent Spalte*	*0*	*1*	*Summe*
0	57<br>25.79<br>50.44<br>52.78	56<br>25.34<br>49.56<br>49.56	113<br>51.13
1	51<br>23.08<br>47.22<br>47.22	57<br>25.79<br>52.78<br>50.44	108<br>48.87
Summe	108<br>48.87	113<br>51.13	221<br>100.00

Statistiken für Tabelle von pad nach PAVK

Statistik	*DF*	*Wert*	*Prob*
Chi-Quadrat	1	0.2292	0.6321
Likelihood-Ratio Chi-Quadrat	1	0.2292	0.6321
Kontinuitätskorr. Chi-Quad.	1	0.1184	0.7308
Mantel-Haenszel Chi-Quadrat	1	0.2281	0.6329
Phi-Koeffizient		0.0322	
Kontingenzkoeffizient		0.0322	
Cramers V		0.0322	

Exakter Test von Fisher	
Zelle (1,1) Häufigkeit (F)	57
Linksseitige Pr <= F	0.7301
Rechtsseitige Pr >= F	0.3654
Tabellenwahrscheinlichkeit (P)	0.0955
Zweiseitige Pr <= P	0.6870

Odds Ratio und relative Risiken			
Statistik	*Wert*	*95% Confidence Limits*	
Odds-Ratio	1.1376	0.6710	1.9287
Relatives Risiko (Spalte 1)	1.0682	0.8151	1.3999
Relatives Risiko (Spalte 2)	0.9390	0.7256	1.2150

Stichprobengröße = 221

Table of pad by CAVK			
pad	CAVK(CAVK)		
Häufigkeit<br>Prozent<br>Prozent Zeile<br>Prozent Spalte	0	1	Summe
0	77<br>34.84<br>68.14<br>57.46	36<br>16.29<br>31.86<br>41.38	113<br>51.13
1	57<br>25.79<br>52.78<br>42.54	51<br>23.08<br>47.22<br>58.62	108<br>48.87
Summe	134<br>60.63	87<br>39.37	221<br>100.00

Statistiken für Tabelle von pad nach CAVK

Statistik	DF	Wert	Prob
Chi-Quadrat	1	5.4610	0.0194
Likelihood-Ratio Chi-Quadrat	1	5.4823	0.0192
Kontinuitätskorr. Chi-Quad.	1	4.8363	0.0279
Mantel-Haenszel Chi-Quadrat	1	5.4362	0.0197
Phi-Koeffizient		0.1572	
Kontingenzkoeffizient		0.1553	
Cramers V		0.1572	

Exakter Test von Fisher	
Zelle (1,1) Häufigkeit (F)	77
Linksseitige Pr <= F	0.9934
Rechtsseitige Pr >= F	0.0138
Tabellenwahrscheinlichkeit (P)	0.0072
Zweiseitige Pr <= P	0.0273

Odds Ratio und relative Risiken			
Statistik	Wert	95% Confidence Limits	
Odds-Ratio	1.9137	1.1073	3.3074

Relatives Risiko (Spalte 1)	1.2911	1.0377	1.6063
Relatives Risiko (Spalte 2)	0.6746	0.4824	0.9435

Stichprobengröße = 221

Table of pad by COPD

pad	*COPD(COPD)*		
Häufigkeit<br>*Prozent*<br>*Prozent Zeile*<br>*Prozent Spalte*	*0*	*1*	*Summe*
0	102<br>46.15<br>90.27<br>50.75	11<br>4.98<br>9.73<br>55.00	113<br>51.13
1	99<br>44.80<br>91.67<br>49.25	9<br>4.07<br>8.33<br>45.00	108<br>48.87
Summe	201<br>90.95	20<br>9.05	221<br>100.00

Statistiken für Tabelle von pad nach COPD

Statistik	*DF*	*Wert*	*Prob*
Chi-Quadrat	1	0.1317	0.7167
Likelihood-Ratio Chi-Quadrat	1	0.1320	0.7164
Kontinuitätskorr. Chi-Quad.	1	0.0165	0.8978
Mantel-Haenszel Chi-Quadrat	1	0.1311	0.7173
Phi-Koeffizient		-0.0244	
Kontingenzkoeffizient		0.0244	
Cramers V		-0.0244	

Exakter Test von Fisher

Zelle (1,1) Häufigkeit (F)	102
Linksseitige Pr <= F	0.4497
Rechtsseitige Pr >= F	0.7241
Tabellenwahrscheinlichkeit (P)	0.1738
Zweiseitige Pr <= P	0.8163

Odds Ratio und relative Risiken

Statistik	Wert	95% Confidence Limits	
Odds-Ratio	0.8430	0.3348	2.1223
Relatives Risiko (Spalte 1)	0.9847	0.9062	1.0700
Relatives Risiko (Spalte 2)	1.1681	0.5040	2.7073

Stichprobengröße = 221

Table of pad by Z_n_Myokardinfarkt

pad	Z_n_Myokardinfarkt(Z.n.Myokardinfarkt)		
Häufigkeit<br>Prozent<br>Prozent Zeile<br>Prozent Spalte	0	1	Summe
0	96<br>43.44<br>84.96<br>50.79	17<br>7.69<br>15.04<br>53.13	113<br>51.13
1	93<br>42.08<br>86.11<br>49.21	15<br>6.79<br>13.89<br>46.88	108<br>48.87
Summe	189<br>85.52	32<br>14.48	221<br>100.00

Statistiken für Tabelle von pad nach Z_n_Myokardinfarkt

Statistik	DF	Wert	Prob
Chi-Quadrat	1	0.0595	0.8072
Likelihood-Ratio Chi-Quadrat	1	0.0596	0.8072
Kontinuitätskorr. Chi-Quad.	1	0.0028	0.9579
Mantel-Haenszel Chi-Quadrat	1	0.0593	0.8077
Phi-Koeffizient		-0.0164	
Kontingenzkoeffizient		0.0164	
Cramers V		-0.0164	

Exakter Test von Fisher	
Zelle (1,1) Häufigkeit (F)	96
Linksseitige Pr <= F	0.4794
Rechtsseitige Pr >= F	0.6677
Tabellenwahrscheinlichkeit (P)	0.1471
Zweiseitige Pr <= P	0.8501

Odds Ratio und relative Risiken			
Statistik	*Wert*	*95% Confidence Limits*	
Odds-Ratio	0.9108	0.4300	1.9293
Relatives Risiko (Spalte 1)	0.9866	0.8852	1.0996
Relatives Risiko (Spalte 2)	1.0832	0.5699	2.0589

Stichprobengröße = 221

Table of pad by Z_n_Apoplex			
pad	*Z_n_Apoplex(Z.n.Apoplex)*		
Häufigkeit<br>*Prozent*<br>*Prozent Zeile*<br>*Prozent Spalte*	*0*	*1*	*Summe*
0	106<br>47.96<br>93.81<br>53.27	7<br>3.17<br>6.19<br>31.82	113<br>51.13
1	93<br>42.08<br>86.11<br>46.73	15<br>6.79<br>13.89<br>68.18	108<br>48.87
Summe	199<br>90.05	22<br>9.95	221<br>100.00

Statistiken für Tabelle von pad nach Z_n_Apoplex

Statistik	*DF*	*Wert*	*Prob*
Chi-Quadrat	1	3.6471	0.0562
Likelihood-Ratio Chi-Quadrat	1	3.7136	0.0540
Kontinuitätskorr. Chi-Quad.	1	2.8392	0.0920
Mantel-Haenszel Chi-Quadrat	1	3.6306	0.0567
Phi-Koeffizient		0.1285	
Kontingenzkoeffizient		0.1274	
Cramers V		0.1285	

Exakter Test von Fisher	
Zelle (1,1) Häufigkeit (F)	106
Linksseitige Pr <= F	0.9844
Rechtsseitige Pr >= F	0.0453
Tabellenwahrscheinlichkeit (P)	0.0297

Zweiseitige Pr <= P	0.0721

Odds Ratio und relative Risiken			
Statistik	*Wert*	*95% Confidence Limits*	
Odds-Ratio	2.4424	0.9547	6.2486
Relatives Risiko (Spalte 1)	1.0894	0.9962	1.1912
Relatives Risiko (Spalte 2)	0.4460	0.1892	1.0514

Stichprobengröße = 221

Table of pad by KHK			
pad	*KHK(KHK)*		
Häufigkeit<br>*Prozent*<br>*Prozent Zeile*<br>*Prozent Spalte*	*0*	*1*	*Summe*
0	56<br>25.34<br>49.56<br>56.57	57<br>25.79<br>50.44<br>46.72	113<br>51.13
1	43<br>19.46<br>39.81<br>43.43	65<br>29.41<br>60.19<br>53.28	108<br>48.87
Summe	99<br>44.80	122<br>55.20	221<br>100.00

Statistiken für Tabelle von pad nach KHK

Statistik	*DF*	*Wert*	*Prob*
Chi-Quadrat	1	2.1196	0.1454
Likelihood-Ratio Chi-Quadrat	1	2.1238	0.1450
Kontinuitätskorr. Chi-Quad.	1	1.7440	0.1866
Mantel-Haenszel Chi-Quadrat	1	2.1100	0.1463
Phi-Koeffizient		0.0979	
Kontingenzkoeffizient		0.0975	
Cramers V		0.0979	

Exakter Test von Fisher	
Zelle (1,1) Häufigkeit (F)	56
Linksseitige Pr <= F	0.9443
Rechtsseitige Pr >= F	0.0932

Tabellenwahrscheinlichkeit (P)	0.0376
Zweiseitige Pr <= P	0.1761

Odds Ratio und relative Risiken			
Statistik	*Wert*	*95% Confidence Limits*	
Odds-Ratio	1.4851	0.8712	2.5315
Relatives Risiko (Spalte 1)	1.2447	0.9246	1.6756
Relatives Risiko (Spalte 2)	0.8381	0.6602	1.0640

Stichprobengröße = 221

Table of pad by AI					
pad	*AI(AI)*				
Häufigkeit<br>*Prozent*<br>*Prozent Zeile*<br>*Prozent Spalte*	*0*	*1*	*2*	*3*	*Summe*
0	44<br>19.91<br>38.94<br>57.89	51<br>23.08<br>45.13<br>46.79	18<br>8.14<br>15.93<br>54.55	0<br>0.00<br>0.00<br>0.00	113<br>51.13
1	32<br>14.48<br>29.63<br>42.11	58<br>26.24<br>53.70<br>53.21	15<br>6.79<br>13.89<br>45.45	3<br>1.36<br>2.78<br>100.00	108<br>48.87
Summe	76<br>34.39	109<br>49.32	33<br>14.93	3<br>1.36	221<br>100.00

Statistiken für Tabelle von pad nach AI

Statistik	*DF*	*Wert*	*Prob*
Chi-Quadrat	3	5.5067	0.1382
Likelihood-Ratio Chi-Quadrat	3	6.6714	0.0831
Mantel-Haenszel Chi-Quadrat	1	1.7402	0.1871
Phi-Koeffizient		0.1579	
Kontingenzkoeffizient		0.1559	
Cramers V		0.1579	
WARNING: 25% der Zellen haben erwartete Häufigkeiten unter 5. Chi-Quadrat ist eventuell kein gültiger Test.			

Exakter Test von Fisher	
Tabellenwahrscheinlichkeit (P)	0.0006

Pr <= P	0.1501

Stichprobengröße = 221

Table of pad by MI					
pad	*MI(MI)*				
Häufigkeit<br>*Prozent*<br>*Prozent Zeile*<br>*Prozent Spalte*	*0*	*1*	*2*	*3*	*Summe*
0	38<br>17.27<br>33.93<br>59.38	43<br>19.55<br>38.39<br>52.44	26<br>11.82<br>23.21<br>43.33	5<br>2.27<br>4.46<br>35.71	112<br>50.91
1	26<br>11.82<br>24.07<br>40.63	39<br>17.73<br>36.11<br>47.56	34<br>15.45<br>31.48<br>56.67	9<br>4.09<br>8.33<br>64.29	108<br>49.09
Summe	64<br>29.09	82<br>37.27	60<br>27.27	14<br>6.36	220<br>100.00
Frequency Missing = 1					

Statistiken für Tabelle von pad nach MI

Statistik	*DF*	*Wert*	*Prob*
Chi-Quadrat	3	4.5834	0.2050
Likelihood-Ratio Chi-Quadrat	3	4.6146	0.2023
Mantel-Haenszel Chi-Quadrat	1	4.5395	0.0331
Phi-Koeffizient		0.1443	
Kontingenzkoeffizient		0.1429	
Cramers V		0.1443	

Exakter Test von Fisher	
Tabellenwahrscheinlichkeit (P)	0.0004
Pr <= P	0.2094

Effektive Stichprobengröße = 220
Häufigkeit Fehlende = 1

Table of pad by TI					
pad	*TI(TI)*				
Häufigkeit<br>*Prozent*<br>*Prozent Zeile*<br>*Prozent Spalte*	*0*	*1*	*2*	*3*	*Summe*

0	45<br>20.36<br>39.82<br>63.38	55<br>24.89<br>48.67<br>45.83	10<br>4.52<br>8.85<br>43.48	3<br>1.36<br>2.65<br>42.86	113<br>51.13
1	26<br>11.76<br>24.07<br>36.62	65<br>29.41<br>60.19<br>54.17	13<br>5.88<br>12.04<br>56.52	4<br>1.81<br>3.70<br>57.14	108<br>48.87
Summe	71<br>32.13	120<br>54.30	23<br>10.41	7<br>3.17	221<br>100.00

Statistiken für Tabelle von pad nach TI

Statistik	*DF*	*Wert*	*Prob*
Chi-Quadrat	3	6.3421	0.0961
Likelihood-Ratio Chi-Quadrat	3	6.4039	0.0935
Mantel-Haenszel Chi-Quadrat	1	4.6031	0.0319
Phi-Koeffizient		0.1694	
Kontingenzkoeffizient		0.1670	
Cramers V		0.1694	
WARNING: 25% der Zellen haben erwartete Häufigkeiten unter 5. Chi-Quadrat ist eventuell kein gültiger Test.			

Exakter Test von Fisher	
Tabellenwahrscheinlichkeit (P)	0.0003
Pr <= P	0.0888

Stichprobengröße = 221

Table of pad by AS			
pad	*AS(AS)*		
Häufigkeit<br>*Prozent*<br>*Prozent Zeile*<br>*Prozent Spalte*	*0*	*1*	*Summe*
0	42<br>19.00<br>37.17<br>60.87	71<br>32.13<br>62.83<br>46.71	113<br>51.13
1	27<br>12.22<br>25.00<br>39.13	81<br>36.65<br>75.00<br>53.29	108<br>48.87
Summe	69<br>31.22	152<br>68.78	221<br>100.00

Statistiken für Tabelle von pad nach AS			
Statistik	*DF*	*Wert*	*Prob*
Chi-Quadrat	1	3.8076	0.0510
Likelihood-Ratio Chi-Quadrat	1	3.8323	0.0503
Kontinuitätskorr. Chi-Quad.	1	3.2620	0.0709
Mantel-Haenszel Chi-Quadrat	1	3.7904	0.0515
Phi-Koeffizient		0.1313	
Kontingenzkoeffizient		0.1301	
Cramers V		0.1313	

Exakter Test von Fisher	
Zelle (1,1) Häufigkeit (F)	42
Linksseitige Pr <= F	0.9822
Rechtsseitige Pr >= F	0.0352
Tabellenwahrscheinlichkeit (P)	0.0174
Zweiseitige Pr <= P	0.0595

Odds Ratio und relative Risiken			
Statistik	*Wert*	*95% Confidence Limits*	
Odds-Ratio	1.7746	0.9946	3.1665
Relatives Risiko (Spalte 1)	1.4867	0.9914	2.2295
Relatives Risiko (Spalte 2)	0.8378	0.7006	1.0018

Stichprobengröße = 221
