# Supplementary material for: Comparative study of pressure (ankle-brachial pressure index) and flow (strain gauge plethysmography and reactive hyperaemia) measurements in diagnosis of peripheral arterial disease in patients with severe aortic stenosis
Source: PLoS One. 2019 Jul 30;14(7):e0220510. doi: 10.1371/journal.pone.0220510 (PMC6667209; doi:10.1371/journal.pone.0220510)
Supplement: S2 Table — (PDF) [file pone.0220510.s002.pdf]

| pad | N | Mittelwert | Std.abw. | Std Err | Minimum | Maximum |
|---|---|---|---|---|---|---|
| 0 | 113 | 3.6018 | 2.5140 | 0.2365 | 0 | 14.0000 |
| 1 | 108 | 3.1194 | 2.0955 | 0.2016 | 0 | 8.6000 |
| Diff (1-2) | | 0.4823 | 2.3190 | 0.3121 | | |

| pad | Methode | Mittelwert | 95% CL Mean | | Std.abw. | 95% CL Std Dev | |
|---|---|---|---|---|---|---|---|
| 0 | | 3.6018 | 3.1332 | 4.0704 | 2.5140 | 2.2234 | 2.8925 |
| 1 | | 3.1194 | 2.7197 | 3.5192 | 2.0955 | 1.8485 | 2.4195 |
| Diff (1-2) | Gepoolt | 0.4823 | -0.1327 | 1.0974 | 2.3190 | 2.1206 | 2.5586 |
| Diff (1-2) | Satterthwaite | 0.4823 | -0.1303 | 1.0949 | | | |

| Methode | Varianzen | DF | t-Wert | Pr > \|t\| |
|---|---|---|---|---|
| Gepoolt | Gleich | 219 | 1.55 | 0.1236 |
| Satterthwaite | Ungleich | 215.06 | 1.55 | 0.1221 |

| Gleichheit der Varianzen | | | | |
|---|---|---|---|---|
| Methode | Num DF | Den DF | F-Wert | Pr > F |
| Folded F | 112 | 107 | 1.44 | 0.0586 |

**Variable: Arterielle_Ruheeinstrom_Indexbe1 (Arterielle_Ruheeinstrom_Indexbein_pre_15s)**

| pad | N | Mittelwert | Std.abw. | Std Err | Minimum | Maximum |
|---|---|---|---|---|---|---|
| 0 | 113 | 3.0168 | 1.9063 | 0.1793 | 0.1000 | 10.5000 |
| 1 | 108 | 2.6991 | 1.6586 | 0.1596 | 0 | 7.0000 |
| Diff (1-2) | | 0.3177 | 1.7896 | 0.2408 | | |

| pad | Methode | Mittelwert | 95% CL Mean | | Std.abw. | 95% CL Std Dev | |
|---|---|---|---|---|---|---|---|
| 0 | | 3.0168 | 2.6615 | 3.3721 | 1.9063 | 1.6860 | 2.1934 |
| 1 | | 2.6991 | 2.3827 | 3.0155 | 1.6586 | 1.4630 | 1.9150 |
| Diff (1-2) | Gepoolt | 0.3177 | -0.1569 | 0.7924 | 1.7896 | 1.6365 | 1.9745 |
| Diff (1-2) | Satterthwaite | 0.3177 | -0.1554 | 0.7909 | | | |

| *Methode* | *Varianzen* | *DF* | *t-Wert* | *Pr > \|t\|* |
|---|---|---|---|---|
| *Gepoolt* | Gleich | 219 | 1.32 | 0.1884 |
| *Satterthwaite* | Ungleich | 217.11 | 1.32 | 0.1870 |

| *Gleichheit der Varianzen* | | | | |
|---|---|---|---|---|
| *Methode* | *Num DF* | *Den DF* | *F-Wert* | *Pr > F* |
| *Folded F* | 112 | 107 | 1.32 | 0.1477 |

***Variable: Arterielle_Ruheeinstrom_Indexbe2 (Arterielle_Ruheeinstrom_Indexbein_pre_30s)***

| *pad* | *N* | *Mittelwert* | *Std.abw.* | *Std Err* | *Minimum* | *Maximum* |
|---|---|---|---|---|---|---|
| *0* | 113 | 3.0381 | 1.8618 | 0.1751 | 0 | 10.0000 |
| *1* | 108 | 2.7074 | 1.7219 | 0.1657 | 0 | 8.0000 |
| *Diff (1-2)* | | 0.3306 | 1.7948 | 0.2415 | | |

| *pad* | *Methode* | *Mittelwert* | *95% CL Mean* | | *Std.abw.* | *95% CL Std Dev* | |
|---|---|---|---|---|---|---|---|
| *0* | | 3.0381 | 2.6910 | 3.3851 | 1.8618 | 1.6466 | 2.1421 |
| *1* | | 2.7074 | 2.3790 | 3.0359 | 1.7219 | 1.5188 | 1.9880 |
| *Diff (1-2)* | *Gepoolt* | 0.3306 | -0.1454 | 0.8066 | 1.7948 | 1.6413 | 1.9802 |
| *Diff (1-2)* | *Satterthwaite* | 0.3306 | -0.1445 | 0.8058 | | | |

| *Methode* | *Varianzen* | *DF* | *t-Wert* | *Pr > \|t\|* |
|---|---|---|---|---|
| *Gepoolt* | Gleich | 219 | 1.37 | 0.1724 |
| *Satterthwaite* | Ungleich | 218.77 | 1.37 | 0.1716 |

| *Gleichheit der Varianzen* | | | | |
|---|---|---|---|---|
| *Methode* | *Num DF* | *Den DF* | *F-Wert* | *Pr > F* |
| *Folded F* | 112 | 107 | 1.17 | 0.4162 |

| ***Variable: Arterielle_Reserve_Indexbein_pre (Arterielle_Reserve_Indexbein_pre_5s)*** |
|---|

| *pad* | *N* | *Mittelwert* | *Std.abw.* | *Std Err* | *Minimum* | *Maximum* |
|---|---|---|---|---|---|---|
| *0* | 113 | 13.5133 | 6.4657 | 0.6082 | 0 | 32.0000 |
| *1* | 108 | 9.1750 | 6.9399 | 0.6678 | 0 | 39.0000 |
| *Diff (1-2)* | | 4.3383 | 6.7016 | 0.9018 | | |

| *pad* | *Methode* | *Mittelwert* | *95% CL Mean* | | *Std.abw.* | *95% CL Std Dev* | |
|---|---|---|---|---|---|---|---|
| *0* | | 13.5133 | 12.3081 | 14.7184 | 6.4657 | 5.7185 | 7.4393 |
| *1* | | 9.1750 | 7.8512 | 10.4988 | 6.9399 | 6.1216 | 8.0126 |
| *Diff (1-2)* | *Gepoolt* | 4.3383 | 2.5609 | 6.1156 | 6.7016 | 6.1284 | 7.3939 |
| *Diff (1-2)* | *Satterthwaite* | 4.3383 | 2.5579 | 6.1186 | | | |

| *Methode* | *Varianzen* | *DF* | *t-Wert* | *Pr > \|t\|* |
|---|---|---|---|---|
| *Gepoolt* | Gleich | 219 | 4.81 | <.0001 |
| *Satterthwaite* | Ungleich | 216.09 | 4.80 | <.0001 |

| *Gleichheit der Varianzen* | | | | |
|---|---|---|---|---|
| *Methode* | *Num DF* | *Den DF* | *F-Wert* | *Pr > F* |
| *Folded F* | 107 | 112 | 1.15 | 0.4592 |

| ***Variable: Arterielle_Reserve_Indexbein_pr1 (Arterielle_Reserve_Indexbein_pre_15s)*** |
|---|

| *pad* | *N* | *Mittelwert* | *Std.abw.* | *Std Err* | *Minimum* | *Maximum* |
|---|---|---|---|---|---|---|
| *0* | 113 | 8.3274 | 4.3762 | 0.4117 | 0 | 23.0000 |
| *1* | 108 | 6.6843 | 4.7522 | 0.4573 | 0 | 27.0000 |
| *Diff (1-2)* | | 1.6432 | 4.5638 | 0.6141 | | |

| *pad* | *Methode* | *Mittelwert* | *95% CL Mean* | | *Std.abw.* | *95% CL Std Dev* | |
|---|---|---|---|---|---|---|---|
| *0* | | 8.3274 | 7.5118 | 9.1431 | 4.3762 | 3.8704 | 5.0351 |
| *1* | | 6.6843 | 5.7778 | 7.5908 | 4.7522 | 4.1919 | 5.4868 |
| *Diff (1-2)* | *Gepoolt* | 1.6432 | 0.4328 | 2.8536 | 4.5638 | 4.1734 | 5.0353 |

| | | | | | | | |
|---|---|---|---|---|---|---|---|
| *Diff (1-2)* | *Satterthwaite* | 1.6432 | 0.4304 | 2.8559 | | | |

| *Methode* | *Varianzen* | *DF* | *t-Wert* | *Pr > \|t\|* |
|---|---|---|---|---|
| *Gepoolt* | Gleich | 219 | 2.68 | 0.0080 |
| *Satterthwaite* | Ungleich | 215.49 | 2.67 | 0.0081 |

| *Gleichheit der Varianzen* | | | | |
|---|---|---|---|---|
| *Methode* | *Num DF* | *Den DF* | *F-Wert* | *Pr > F* |
| *Folded F* | 107 | 112 | 1.18 | 0.3887 |

**Variable: Arterielle_Reserve_Indexbein_pr2 (Arterielle_Reserve_Indexbein_pre_25s)**

| *pad* | *N* | *Mittelwert* | *Std.abw.* | *Std Err* | *Minimum* | *Maximum* |
|---|---|---|---|---|---|---|
| *0* | 112 | 6.3482 | 3.8897 | 0.3675 | 1.0000 | 20.0000 |
| *1* | 107 | 5.3458 | 3.8951 | 0.3766 | 0 | 16.0000 |
| *Diff (1-2)* | | 1.0024 | 3.8923 | 0.5262 | | |

| *pad* | *Methode* | *Mittelwert* | *95% CL Mean* | | *Std.abw.* | *95% CL Std Dev* | |
|---|---|---|---|---|---|---|---|
| *0* | | 6.3482 | 5.6199 | 7.0765 | 3.8897 | 3.4384 | 4.4784 |
| *1* | | 5.3458 | 4.5992 | 6.0923 | 3.8951 | 3.4339 | 4.5005 |
| *Diff (1-2)* | *Gepoolt* | 1.0024 | -0.0347 | 2.0395 | 3.8923 | 3.5581 | 4.2965 |
| *Diff (1-2)* | *Satterthwaite* | 1.0024 | -0.0347 | 2.0395 | | | |

| *Methode* | *Varianzen* | *DF* | *t-Wert* | *Pr > \|t\|* |
|---|---|---|---|---|
| *Gepoolt* | Gleich | 217 | 1.91 | 0.0581 |
| *Satterthwaite* | Ungleich | 216.52 | 1.91 | 0.0581 |

| *Gleichheit der Varianzen* | | | | |
|---|---|---|---|---|
| *Methode* | *Num DF* | *Den DF* | *F-Wert* | *Pr > F* |
| *Folded F* | 106 | 111 | 1.00 | 0.9874 |

| *Variable: Arterielle_Reserve_Indexbein_pr3 (Arterielle_Reserve_Indexbein_pre_35s)* |
|---|

| *pad* | *N* | *Mittelwert* | *Std.abw.* | *Std Err* | *Minimum* | *Maximum* |
|---|---|---|---|---|---|---|
| *0* | 112 | 5.0893 | 3.4290 | 0.3240 | 0 | 19.0000 |
| *1* | 107 | 4.6907 | 3.3556 | 0.3244 | 0 | 14.0000 |
| *Diff (1-2)* | | 0.3986 | 3.3933 | 0.4587 | | |

| *pad* | *Methode* | *Mittelwert* | *95% CL Mean* | | *Std.abw.* | *95% CL Std Dev* | |
|---|---|---|---|---|---|---|---|
| *0* | | 5.0893 | 4.4472 | 5.7313 | 3.4290 | 3.0311 | 3.9479 |
| *1* | | 4.6907 | 4.0475 | 5.3338 | 3.3556 | 2.9583 | 3.8771 |
| *Diff (1-2)* | *Gepoolt* | 0.3986 | -0.5055 | 1.3027 | 3.3933 | 3.1019 | 3.7457 |
| *Diff (1-2)* | *Satterthwaite* | 0.3986 | -0.5050 | 1.3023 | | | |

| *Methode* | *Varianzen* | *DF* | *t-Wert* | *Pr > \|t\|* |
|---|---|---|---|---|
| *Gepoolt* | Gleich | 217 | 0.87 | 0.3858 |
| *Satterthwaite* | Ungleich | 216.87 | 0.87 | 0.3856 |

| *Gleichheit der Varianzen* | | | | |
|---|---|---|---|---|
| *Methode* | *Num DF* | *Den DF* | *F-Wert* | *Pr > F* |
| *Folded F* | 111 | 106 | 1.04 | 0.8234 |

| *Variable: Arterielle_Reserve_Indexbein_pr4 (Arterielle_Reserve_Indexbein_pre_45s)* |
|---|

| *pad* | *N* | *Mittelwert* | *Std.abw.* | *Std Err* | *Minimum* | *Maximum* |
|---|---|---|---|---|---|---|
| *0* | 113 | 4.9912 | 3.4187 | 0.3216 | 0 | 19.0000 |
| *1* | 108 | 4.3602 | 3.3118 | 0.3187 | 0 | 17.0000 |
| *Diff (1-2)* | | 0.6310 | 3.3669 | 0.4531 | | |

| *pad* | *Methode* | *Mittelwert* | *95% CL Mean* | *Std.abw.* | *95% CL Std Dev* |
|---|---|---|---|---|---|

| | | | | | | | |
|---|---|---|---|---|---|---|---|
| *0* | | 4.9912 | 4.3539 | 5.6284 | 3.4187 | 3.0236 | 3.9335 |
| *1* | | 4.3602 | 3.7284 | 4.9919 | 3.3118 | 2.9213 | 3.8238 |
| *Diff (1-2)* | *Gepoolt* | 0.6310 | -0.2620 | 1.5239 | 3.3669 | 3.0789 | 3.7147 |
| *Diff (1-2)* | *Satterthwaite* | 0.6310 | -0.2613 | 1.5233 | | | |

| *Methode* | *Varianzen* | *DF* | *t-Wert* | *Pr > \|t\|* |
|---|---|---|---|---|
| *Gepoolt* | Gleich | 219 | 1.39 | 0.1651 |
| *Satterthwaite* | Ungleich | 218.96 | 1.39 | 0.1648 |

| *Gleichheit der Varianzen* | | | | |
|---|---|---|---|---|
| *Methode* | *Num DF* | *Den DF* | *F-Wert* | *Pr > F* |
| *Folded F* | 112 | 107 | 1.07 | 0.7415 |

***Variable: Peak_Flow_Indexbein_pre (Peak_Flow_Indexbein_pre)***

| *pad* | *N* | *Mittelwert* | *Std.abw.* | *Std Err* | *Minimum* | *Maximum* |
|---|---|---|---|---|---|---|
| *0* | 113 | 6.1504 | 4.1731 | 0.3926 | 5.0000 | 35.0000 |
| *1* | 108 | 11.7593 | 9.8426 | 0.9471 | 5.0000 | 45.0000 |
| *Diff (1-2)* | | -5.6088 | 7.4992 | 1.0092 | | |

| *pad* | *Methode* | *Mittelwert* | *95% CL Mean* | | *Std.abw.* | *95% CL Std Dev* | |
|---|---|---|---|---|---|---|---|
| *0* | | 6.1504 | 5.3726 | 6.9283 | 4.1731 | 3.6908 | 4.8014 |
| *1* | | 11.7593 | 9.8817 | 13.6368 | 9.8426 | 8.6821 | 11.3640 |
| *Diff (1-2)* | *Gepoolt* | -5.6088 | -7.5977 | -3.6199 | 7.4992 | 6.8578 | 8.2740 |
| *Diff (1-2)* | *Satterthwaite* | -5.6088 | -7.6354 | -3.5822 | | | |

| *Methode* | *Varianzen* | *DF* | *t-Wert* | *Pr > \|t\|* |
|---|---|---|---|---|
| *Gepoolt* | Gleich | 219 | -5.56 | <.0001 |
| *Satterthwaite* | Ungleich | 142.9 | -5.47 | <.0001 |

| *Gleichheit der Varianzen* | | | | |
|---|---|---|---|---|
| *Methode* | *Num DF* | *Den DF* | *F-Wert* | *Pr > F* |

| | | | | |
|---|---|---|---|---|
| *Folded F* | 107 | 112 | 5.56 | <.0001 |
